# Is the beta3-adrenoceptor (ADRB3) a potential target for uterorelaxant drugs?

**DOI:** 10.1186/1471-2393-7-S1-S14

**Published:** 2007-06-01

**Authors:** Marc Bardou, Céline Rouget, Michèle Breuiller-Fouché, Catherine Loustalot, Emmanuel Naline, Paul Sagot, René Frydman, Esteban J Morcillo, Charles Advenier, Marie-Josèphe Leroy, John J Morrison

**Affiliations:** 1 Laboratoire de Physiologie et Pharmacologie Cardiovasculaires Expérimentales (LPPCE), Faculté de Médecine, Université de Bourgogne, 7 Bd Jeanne d'Arc, BP 87900, 21079 Dijon Cedex, France; 2 UPRES EA220 – Pharmacologie, UFR Biomédicale des Saints Pères, Université Paris 5, 45 rue des Saints Pères, 75006 Paris, France; 3 Unité INSERM U767, Faculté des Sciences Pharmaceutiques et Biologiques, Université René Descartes, Université Paris 5, 75006 Paris, France; 4 Department of Gynaecology and Obstetrics, CHU du Bocage, 21 Bd de Lattre de Tassigny, BP 1542, 21000 Dijon, France; 5 Department of Gynaecology and Obstetrics, Antoine Béclère Hospital, 157 rue de la Porte de Trivaux, 92141 Clamart Cedex, France; 6 Department of Pharmacology, University of Valencia, Av. Blasco Ibanez 17, 46010 Valencia, Spain; 7 Department of Gynaecology and Obstetrics, National University of Ireland Galway, Clinical Science Institute, University College Hospital Galway, Galway, Ireland

## Abstract

The management of premature birth still remains unsatisfactory. Since the relative lack of efficiency and/or safety of current tocolytic agents have been highlighted, it is necessary to develop new uterorelaxant drugs deprived of important maternal and foetal side effects. Our work reported in this review focuses on a potential new target for tocolytic drugs, the β_3_-adrenoceptor (ADRB3). This third type of ADRB is shown to be present and functional in human myometrium. We demonstrated that ADRB3 agonists are able to inhibit *in-vitro *spontaneous contractions of myometrial strips, *via *a cyclic AMP-mediated pathway. Furthermore, we established that ADRB3 is the predominant subtype over the ADRB2 in human myometrium and that its expression is increased in near-term myometrium, compared to non-pregnant myometrium. Finally, we reported that contrary to ADRB2, the human myometrial ADRB3 is resistant to long-term agonist-induced desensitisation. These compelling data confirm the clinical potential interest of ADRB3 agonists in the pharmacological management of preterm labour.

## Background and aims

The incidence of premature birth has risen over the past 15 years, mainly because of medically induced births, yet spontaneous preterm delivery rate continues its steady rise and remains relatively high in developed countries, despite preventative measures [1-3]. Approximately 6 to 12% of all pregnancies end up prematurely in Western countries, and the delivery of infants at preterm periods of gestation, and the clinical implications thereof, constitutes a major challenge for neonatologists [4]. The morbidity and mortality associated with preterm delivery outweigh all other clinical problems in obstetric practice [5-7]. Indeed preterm birth is the most frequent cause of infant death in the United States, accounting for at least one third of infant deaths [8]. Several stimulatory and inhibitory pathways regulate the balance of uterine quiescence and contractile activity during pregnancy, but the specific changes that govern the switch between these opposing functional states are still poorly understood.

These data explain that a logical research pursuit has been that of development of pharmacological agents that inhibit uterine contractions and ideally terminate the labor process, or delay delivery until gestation is further advanced.

Unfortunately the efficacy of current pharmacological treatments for the management of preterm labor is regularly questioned. Among these treatments, β_2_-adrenoceptor (ADRB2) agonists are becoming less used worldwide as tocolytic agents because of important maternal and fetal side effects. For these reasons, and the lack of efficacy, much research in the last decade has focused on the development of novel agents, such as calcium channel blockers and oxytocin antagonists [9-12]. In countries outside of the United States, these agents have now largely displaced the use of ADRB2 agonists as tocolytic compounds, but the data to support their use, in terms of clinical efficacy, are lacking.

Although ADRB were originally sub-classified into ADRB1 and ADRB2 [13], another subtype, the ADRB3-subtype, has been since reported [14]. The ADRB3 shares 40 to 50% amino acid sequence identity with ADRB1 and ADRB2. It has been shown to mediate lipolysis in white adipose tissue and thermogenesis in brown adipose tissue [15,16], and to inhibit the contractile activity of ileum and colon [17].

In the heart, ADRB pathways are the primary means of increasing cardiac performance in response to acute or chronic stress. The presence and function of the ADRB3 in the cardiovascular system is a conflicting debate. Indeed it has recently been suggested that ADRB3 are expressed in the endothelium of human coronary resistance arteries, or internal mammary arteries, and mediate adrenergic vasodilatation [18,19].

Myometrium has always been described as expressing predominantly? ADRB2 [20-23] and the presence of ADRB3 had never been studied prior to our investigations presented here.

These works aimed to assess the presence and function of ADRB3 in human myometrium and to assess the influence of pregnancy in its expression as well as its ability to resist to agonist-induced desensitization.

## Results

### The ADRB3 is present and functional in human near term myometrium

**Figure 1:**
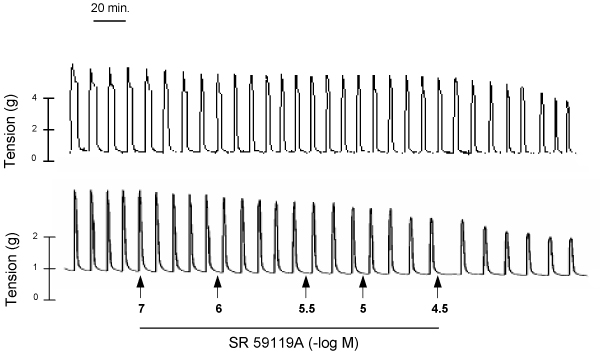
Representative recording of the effect of SR 59119A on spontaneous contractions of human non-pregnant (upper trace) and near-term (lower trace) myometrial strips.

**Figure 2:**
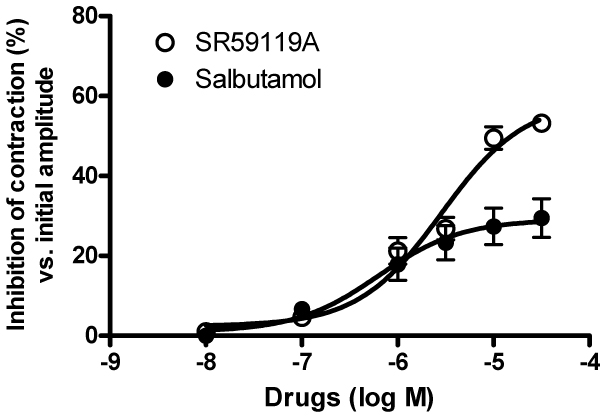
Concentration-response curve for SR59119 and Salbutamol (ADRB3 and ADRB2 agonists respectively) in human near term myometrium.

**Figure 3:**
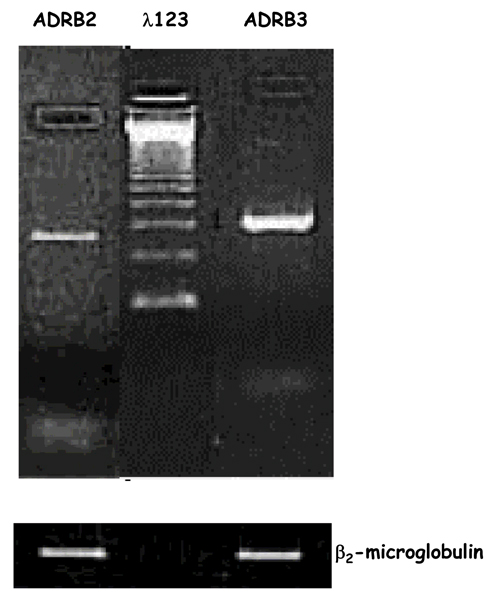
**Representative RT-PCR analysis of the mRNA steady-state levels for ADRB2 and ADRB3 in human pregnant myometrium**. The 123-bp DNA ladder (λ 123) was used to estimate the size of the PCR products, and beta2 microglobulin expression was used as a standard reference.

Our first approach consisted of investigating the presence of the ADRB3 in human near-term myometrium [24]. *In-vitro *functional studies were performed to compare the effects of ADRB2 and ADRB3 agonists on spontaneous contractions. SR 59119A, a selective ADRB3 agonist, produced a concentration-dependent inhibition of spontaneous contractions (Figure 1), with a maximal effect of 52 ± 7% compared with 27 ± 6% for salbutamol (Figure 2). The pharmacological evidence of the ADRB3-mediated nature of this effect was supported by the use of ADRB antagonists. Indeed, propranolol, at a concentration that blocks ADRB1&2 but not ADRB3 [25], and ICI 118551, an ADRB2 antagonist, did not affect the SR 59119A-induced relaxation whereas the ADRB3 antagonist [26], SR 59230A, significantly reduced the maximal effect of SR 59119A. To assess the nature of the second messenger involved in the uterorelaxant effect of SR 59119A, we measured the production of cAMP and cGMP after a stimulation of myometrial strips with SR 59119A or salbutamol. Both SR 59119A and salbutamol induced a significant increase in cAMP levels, but not of cGMP. The production of cAMP was antagonized by SR 59230A but not by propranolol for SR 59119A, providing confirmation of the ADRB3-mediated nature of this stimulation. Using an RT-PCR approach we found that mRNA expression was detected, as an expected 650 bp fragment, in human myometrium (Figure 3). Hybridization to human *ADRB3 *cDNA confirmed the identity of the amplified products.

A somewhat similar pharmacological study was published by Dennedy *et al. *in 2001 [27], on this occasion comparing the functional effects of the ADRB3 agonist BRL 37344, with those of the commonly used ADRB2 agonist, ritodrine, on human pregnant myometrial spontaneous or oxytocin-induced contractility. Mean maximal inhibition of contractility values of approximately 60% were reported for both compounds, at tissue bath concentrations in the 10^-5 ^molar range. There was no difference between calculated pD_2 _values, or mean maximal inhibition achieved, for both compounds (Table 1).

As a logical follow on to the above findings, Dennedy *et al. *[28] further evaluated the selectivity of ADRB3 agonists in human myometrium. Using bupranolol (an ADRB1, ADRB2, and ADRB3 antagonist), propranolol (an ADRB1 and ADRB2 antagonist) and butoxamine (an ADRB2 antagonist), it was demonstrated that the myorelaxant effect of BRL 37344 was mediated via the ADRB3, although ritodrine may exert its effects via ADRB1, ADRB2 and ADRB3.

### The ADRB3 is predominant over the ADRB2 and its expression is increased during pregnancy

Whereas the existence of the third ADRB subtype had not been shown yet formally, we previously described that ADRB2 were predominant (65%) over ADRB1 in the human myometrium and that the number of ADRB was diminished at the end of pregnancy [20]. In the light of our new results, we wanted to assess the influence of pregnancy on the function and the expression of the myometrial ADRB3-subtype [29]. To this end, we performed *in-vitro *studies in human non-pregnant and near-term myometrium and found that SR 59119A inhibited spontaneous contractions of pregnant myometrial strips in a greater extent than that of non-pregnant myometrial strips (E_max _= 61 ± 5% *vs. *44 ± 5% for pregnant and non-pregnant myometrium respectively). On the contrary, salbutamol was less efficient in pregnant than in non-pregnant myometrial strips (E_max _= 29 ± 4% *vs. *54 ± 8%). Binding competition studies, transcripts and protein analysis were then performed to determine whether any changes in the number of ADRB2 and ADRB3 binding sites and in the transcript and protein expression might explain the observed change in the contractile response between non-pregnant and pregnant myometrium to ADRB2 and ADRB3 agonists. Although two populations of binding sites corresponding to ADRB2 and ADRB3 were identified in both non-pregnant and pregnant myometrium, we found a clear predominance of the ADRB3 subtype, a result that contrasts with conventional belief. Moreover, ADRB3 binding sites were 2-fold more numerous at the end of pregnancy, whereas, according to the work of Gsell *et al. *[30], we found no modification in the number of ADRB2 binding sites in non-pregnant compared to pregnant human myometrium.

Densitometric analysis of the RT-PCR experiments indicated a slight decrease in the signal for ADRB2 mRNA (-27.4%) in near-term myometrium comparatively to non-pregnant tissue whereas the signal for ADRB3 mRNA was increased in pregnant myometrium (+66.3%).

**Figure 4:**
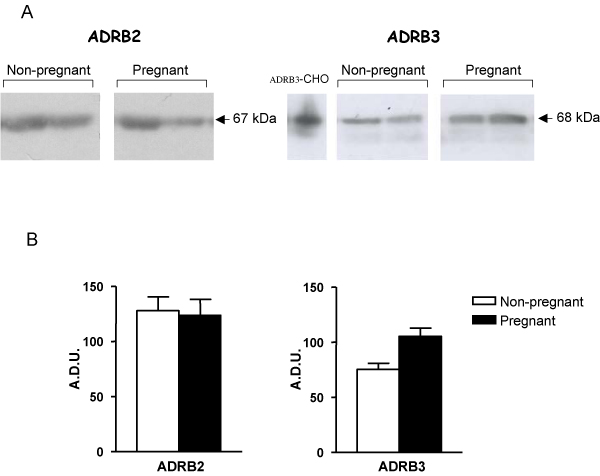
**ADRB2 and ADRB3 immunoreactive proteins expression in myometrium of non-pregnant and pregnant women**. **(A) **Representative western blot analysis of ADRB2- and ADRB3 expression in plasma membranes from myometrium of non-pregnant and pregnant women. Expected sizes are 67 kDa for ADRB2 and 68 kDa for ADRB3. These experiments were performed on myometrium from five non-pregnant and five pregnant women. Homogenate of Chinese Hamster Ovary cells transfected with the human ADRB3 (ADRB3-CHO) was used as positive control. **(B) **Analysis of the expression of ADRB2 and ADRB3 immunoreactive proteins in myometrium of non-pregnant and pregnant women. A.D.U. represents the intensity of the bands evaluated by densitometry. Each bar represents the mean ± s.e.m. from five different non-pregnant and five different pregnant women. Figure reproduced from the paper by Rouget *et al. *[29].

In agreement with Chanrachakul *et al. *[31], densitometric immunoblot analysis indicated no change in the intensity of the signal for the *ADRB2 *in the myometrium of near-term compared to non-pregnant women. At the opposite, an increase (+40%) in the intensity of the signal for the *ADRB3 *protein was observed in pregnant compared with non-pregnant myometrium (Figure 4).

### Contrary to the ADRB2, the ADRB3 is resistant to agonist-induced desensitization

Besides their maternal and fetal side effects, ADRB2 agonists display a lack of efficacy in late pregnancy or after a chronic exposure [32,33]. This loss of efficacy, also called desensitization, may be partly explained by a decreased number of ADRB2 at the end of pregnancy, as well as by an impairment of the coupling between the receptor and its effector, the adenylyl cyclase. ADRB3 was suggested to be less prone than ADRB2 to desensitisation [34]. This particularity may be due to a lack of recognition sites in ADRB3 for the cyclic AMP-dependent protein kinase and for the G-protein coupled receptor kinase implicated in the desensitization process of ADRB2. In order to confirm this hypothesis in human near-term myometrium, we investigated the effects of a long-term (15 h) exposure of myometrial strips to SR 59119A or to salbutamol on the inhibition of spontaneous contractile activity and on the cAMP production induced by these agonists [35]. In our experimental conditions, we demonstrated that ADRB2 undergoes functional desensitization after long-term exposure to salbutamol, since salbutamol concentration-response curve (CRC) was shifted by a 15 h salbutamol pre-incubation with a significant difference in -logEC_20 _values (6.31 ± 0.13 *vs. *5.58 ± 0.24, for control and 15 h salbutamol pre-treatment respectively, P < 0.05). Additionally a 15 h exposure of myometrial strips to salbutamol significantly reduced the salbutamol-induced (0.60 ± 0.26 *vs. *1.54 ± 0.24 pmol.mg^-1 ^protein, P < 0.05) cAMP production. In contrast, a sustained stimulation by SR 59119A did not modify its subsequent functional effects.

A 15 h salbutamol exposure of myometrial strips significantly reduced the ADRB2 but not the ADRB3 binding sites density whereas no decrease in the number of ADRB2 and ADRB3 binding sites was observed after a 15 h SR 59119A treatment. RT-PCR experiments and PDE4 activity measurement were performed in order to assess some of the mechanisms that could be involved in desensitization process and we found that neither *ADRB2 *and *ADRB3 *mRNA expression levels nor PDE4 activity were affected by salbutamol or SR 59119A treatments.

## Discussion and conclusion

Taken altogether our results indicate that ADRB3 is: i) present and functional in human near term myometrium, ii) predominant over ADRB2 in human myometrium and over-expressed in pregnancy, and iii) resistant to the long-term agonist-induced desensitization in human myometrium at the end of pregnancy.

The presence of ADRB3 in human myometrium was confirmed in a work by Hakak *et al. *[36] who compared the ADRB3 expression in Human and mouse. In this paper the two key messages were as follows: (i) ADRB3 expression differs strongly between Human and Mouse myometrium and (ii) human ADRB3 is highly expressed in placenta, ovaries and myometrium (the relative level of expression being as follow: placenta = bladder > aorta > ovary > myometrium)

To conclude, our work strongly suggests the ADRB3 as a potential target for tocolytic agents. The greater relaxant effectiveness of ADRB3 agonists compared to ADRB2 mimetics, as well as the resistance of ADRB3 to sustained agonist-induced desensitization strengthens this concept. We previously mentioned maternal and fetal cardiovascular side effects as one of the major limitations for ADRB2 agonists' use in clinical practice. There is strong evidence to suggest a favorable safety profile, probably better than that of ADRB2 agonists and calcium channels antagonists. Indeed clinical trials, that were designed to assess the effect of ADRB3 agonists on human metabolism, did not reveal any significant change in heart rate, blood pressure, plasma glucose, insulin or potassium levels [37]. Although the therapeutic efficiency of the ADRB3 agonists remains to be established, we believe that ADRB3 agonists may have considerable future pharmaceutical implications in the clinical management of preterm labor.

## List of abbreviations

ADRB, beta-adrenergic receptor; CAMP, cyclic adenosine monophosphate; CGMP, cyclic guanosine monophosphate; CRC, concentration response curve; Emax, maximal efficacy; PDE, phosphodiesterases; RT-PCR, real time polymerase chain reaction.

## Competing interests

The authors declare that they have no competing interests.

## Figures and Tables

**Table 1:** Emax, pD2 and intrinsic activity (IA) for the different ADRB2 or ADRB3 agonists presented in this paper

**Compounds**	**Emax**	**pD2**	**IA**
Isoprenaline	58% ± 9%	7.32 ± 0.62	100% (ref)
Salbutamol	27% ± 6%	6.0 ± 0.48	48% ± 7%
Ritodrine	62% ± 5%	7.26 ± 0.48	107% ± 6%
BRL 37344	60% ± 4%	7.64 ± 0.53	103% ± 4%
SR59119	52% ± 7%	5.65 ± 0.14	90% ± 7%

Emax is expressed as a percentage of inhibition compared with initial amplitude of the contractions. The intrinsic activity (%IA) relative to the maximal effect of (-)-isoproterenol indicate the inhibition of the initial amplitude of spontaneous contractions.
